# Water-Processed Ultrathin Crystalline Indium–Boron–Oxide Channel for High-Performance Thin-Film Transistor Applications

**DOI:** 10.3390/nano12071125

**Published:** 2022-03-29

**Authors:** Wangying Xu, Tao Peng, Yujia Li, Fang Xu, Yu Zhang, Chun Zhao, Ming Fang, Shun Han, Deliang Zhu, Peijiang Cao, Wenjun Liu, Youming Lu

**Affiliations:** 1 Guangdong Research Center for Interfacial Engineering of Functional Materials, Shenzhen Key Laboratory of Special Functional Materials, College of Materials Science and Engineering, Shenzhen University, Shenzhen 518000, China; ptszu0311@163.com (T.P.); 812103935@163.com (Y.L.); m.fang@szu.edu.cn (M.F.); hsdf52690@126.com (S.H.); pjcao@szu.edu.cn (P.C.); liuwj@szu.edu.cn (W.L.); ymlu@szu.edu.cn (Y.L.); 2 Center for Advanced Material Diagnostic Technology, Shenzhen Key Laboratory of Ultraintense Laser and Advanced Material Technology, College of Engineering Physics, Shenzhen Technology University, Shenzhen 518118, China; 3 Department of electronic and Communication Engineering, Shenzhen Polytechnic, Shenzhen 518055, China; zhangyu18@szpt.edu.cn; 4 Department of Electrical and Electronic Engineering, Xi’an Jiaotong-Liverpool University, Suzhou 215123, China; chun.zhao@xjtlu.edu.cn

**Keywords:** In-B-O, thin-film transistors, crystalline, water processed, ultrathin, atomically smooth, ZrO_2_ dielectric

## Abstract

Thin-film transistors (TFTs) made of solution-processable transparent metal oxide semiconductors show great potential for use in emerging large-scale optoelectronics. However, current solution-processed metal oxide TFTs still suffer from relatively poor device performance, hindering their further advancement. In this work, we create a novel ultrathin crystalline indium–boron–oxide (In-B-O) channel layer for high-performance TFTs. We show that high-quality ultrathin (~10 nm) crystalline In-B-O with an atomically smooth nature (RMS: ~0.15 nm) could be grown from an aqueous solution via facile one-step spin-coating. The impacts of B doping on the physical, chemical and electrical properties of the In_2_O_3_ film are systematically investigated. The results show that B has large metal–oxide bond dissociation energy and high Lewis acid strength, which can suppress oxygen vacancy-/hydroxyl-related defects and alleviate dopant-induced carrier scattering, resulting in electrical performance improvement. The optimized In-B-O (10% B) TFTs based on SiO_2_/Si substrate demonstrate a mobility of ~8 cm^2^/(V s), an on/off current ratio of ~10^6^ and a subthreshold swing of 0.86 V/dec. Furthermore, by introducing the water-processed high-K ZrO_2_ dielectric, the fully aqueous solution-grown In-B-O/ZrO_2_ TFTs exhibit excellent device performance, with a mobility of ~11 cm^2^/(V s), an on/off current of ~10^5^, a subthreshold swing of 0.19 V/dec, a low operating voltage of 5 V and superior bias stress stability. Our research opens up new avenues for low-cost, large-area green oxide electronic devices with superior performance.

## 1. Introduction

Transparent metal oxide semiconductors have attracted worldwide attention for thin-film transistor (TFTs) applications due to their superior properties, including good transparency, high electron mobility, reasonable electrical stability and good uniformity [1,2,3,4,5,6,7,8,9,10]. Among them, In_2_O_3_ is widely studied due to its large vacant s-orbital overlap of adjacent cations, which can provide high electron mobility [2,8]. However, it is difficult to turn off the intrinsic In_2_O_3_ TFTs due to the high carrier density, which is caused by the large number of oxygen vacancies. Furthermore, the instability issue of pristine In_2_O_3_ TFTs would also inhibit its practical applications in Active Matrix Liquid Crystal Displays (AMLCDs) and Active Matrix Organic Light-Emitting Diodes (AMOLEDs) [1,2].

Doping with metal cations has proven to be an effective approach to improve the electrical performance of pristine In_2_O_3_ devices [11,12,13,14,15,16,17]. According to previous research [18], ideal dopants should have high Lewis acid strength (L = Z/r^2^ − 7.7χz + 8.0, where r is ionic radius of doped cation, z denotes effective nuclear charge number and χz represents the electronegativity of the element) and high dopant–oxygen bond dissociation energy. The high dopant–oxygen bond dissociation energy would effectively suppress oxygen vacancies, while high Lewis acid strength could reduce carrier scattering and maintain the In_2_O_3_-based material mobility at a relatively high level [18]. The Lewis acid strength and metal–oxide bonding dissociation energy of commonly used dopants are summarized in Table 1. It is worth noting that among potential dopants, boron (B) is an ideal dopant owing to its high Lewis acid strength, mainly due to the small ionic radius of B (~0.023 nm) and high B-O bonding dissociation energy (808.8 KJ/mol), which is conducive to reducing carrier concentration and realizing the creation of high-performance metal oxide devices [18].

Parthiban et al. reported on B-doped In-Zn-O TFTs with a mobility of 9.6 cm^2^/(V s) and Stewart et al. demonstrated B-doped In_2_O_3_ TFTs with a mobility of 20 cm^2^/(V s) [14,17]. Unfortunately, both these B-doped metal oxides were prepared using a vacuum-based deposition approach, which is not good for potential low-cost applications. Park et al. demonstrated solution-processed B-doped In-Zn-O TFTs with a mobility of 7.9 cm^2^/(V s) [19]. However, they did not vary the B doping ratio of the B-In-Zn-O semiconductor and the role of B doping is not revealed. In 2018, Zhang et al. carried out the first systematic study on solution-processed, B-doped In_2_O_3_ TFTs, showing an optimized mobility of 8 cm^2^/(V s) (6% B doping) on a SiO_2_/Si substrate [13]. However, there are still some shortcomings for solution-based In-B-O TFTs that require further investigations. First, an organic solvent is used for In-B-O preparation, which is unsafe and harmful to human beings and the environment. Secondly, repeated spin-coating is needed in the fabrication of In-B-O thin film, which will introduce defect states in the interface and also increase process complexity. Thirdly, combustion chemistry was used in this study, which is a difficult process to control. Fourthly, amorphous In-B-O was reported in Zhang’s research. Recent studies have suggested that a crystalline oxide semiconductor may be better for creating high-mobility and high-stability electronic devices owing to the more effective conduction paths and fewer defect states [20]. Therefore, it will be interesting if crystalline In-B-O could be realized via simple solution processing.

In the current work, we introduce a novel aqueous route to produce an In-B-O thin film, which is considered to be safer, healthier and more environmentally friendly. Moreover, the aqueous precursor is insensitive to ambient humidity and, hence, is easy to handle. Water is also an excellent solvent, since it contains no organic residues that need to be removed. As a result, we found that high-quality crystalline In-B-O could be realized using a facile, one-step, spin-coated aqueous process [21]. The effects of B doping on the physical, chemical and electrical properties of the In_2_O_3_ films are comprehensively examined. It is revealed that B has a strong bonding energy with O and high Lewis acid strength, which could inhibit oxygen vacancy/hydroxyl defect formation and weaken carrier scattering. Meanwhile, the fabricated crystalline In-B-O shows an ultrathin (~10 nm) and atomically smooth nature (RMS: ~0.15 nm), which is good for device applications. The optimized In-B-O TFTs based on SiO_2_/Si substrate exhibits a mobility of ~8 cm^2^/(V s), an on/off current ratio of ~10^6^ and a subthreshold swing of 0.86 V/dec. Furthermore, we realize an all-water-processed high-performance oxide TFTs that combines a In-B-O channel and a ZrO_2_ dielectric with a mobility of ~11 cm^2^/(V s), an on/off current of ~10^5^, a subthreshold swing of 0.19 V/dec, a low operating voltage of 5 V and superior bias stress stability.

## 2. Experimental

Precursor preparation: All the chemicals were obtained from Sigma Aldrich. Indium nitrate hydrate (In(NO_3_)_3_·xH_2_O, 99.9% trace metals basis) and boric acid (H_3_BO_3_, 99.9% trace metals basis) were dissolved in deionized (DI) water and stirred for 1 h at room temperature to prepare the indium–boron–oxide (In-B-O) precursor solution. The solution’s concentration was maintained at 0.2 M. The mole ratios of B/(B + In) were varied from 0% to 15%. To prepare the ZrO_2_ dielectric layer, we dissolved 1 M zirconium nitrate (Zr(NO_3_)_4_·5H_2_O, 99.9% trace metals basis) in DI water to make the ZrO_2_ precursor solution. Then, the ZrO_2_ precursor was vigorously stirred for 6 h at room temperature. All precursor solutions were filtered through a 0.22 μm syringe filter.

Thin films and devices fabrications: The heavily doped Si wafers (P^++^ Si) with thermally grown SiO_2_ (100 nm) were used as substrates and dielectric layers for In-B-O/SiO_2_ TFTs. The substrates were ultrasonically cleaned with acetone, alcohol and DI water for 15 min and then treated with oxygen plasma for 10 min. For In-B-O/ZrO_2_ TFTs, the ZrO_2_ dielectric was fabricated by spin-coating the ZrO_2_ precursor on a P^++^ Si substrate at 4000 rpm for 30 s, followed by 350 °C annealing for 1 h. Next, the In-B-O precursor solution was spun on the SiO_2_/P^++^ Si or ZrO_2_/P^++^ Si substrates and subsequently annealed at 350 °C for 1 h in air. Finally, 90 nm-thick Al electrodes were thermally evaporated through shadow masks with channel dimensions of 100 μm (L) × 1500 μm (W) to finish the In-B-O/SiO_2_ and In-B-O/ZrO_2_ TFTs fabrications.

Characterization: The crystal structures of In-B-O thin films were analyzed via grazing incidence X-ray diffraction (GIXRD, Rigaku SmartLab). Atomic force microscopy (AFM, Bruker Dimension ICON) and transmission electron microscopy (TEM, Tecnai G2 F20 S-TWIN) were used to observe the In-B-O microstructures and morphologies. The chemical bonding states of In-B-O were analyzed via X-ray photoelectron spectroscopy (XPS, Thermo Microlab 350). The optical transmittances of In-B-O thin films were recorded using UV-vis spectroscopy (PerkinElmer Lambda 950) from 200 to 800 nm. The electrical measurements of the TFTs were analyzed with a semiconductor device analyzer (Keithley 2614B).

## 3. Results and Discussion

To explore the role of B doping on In-B-O thin films, systematical investigations with GIXRD, TEM, AFM, XPS and UV-vis spectroscopy were carried out. Next, In-B-O TFTs with different B doping ratios were investigated. Finally, the optimized In-B-O was integrated with a ZrO_2_ high-K dielectric to further improve the performance of the device.

**Figure 1:**
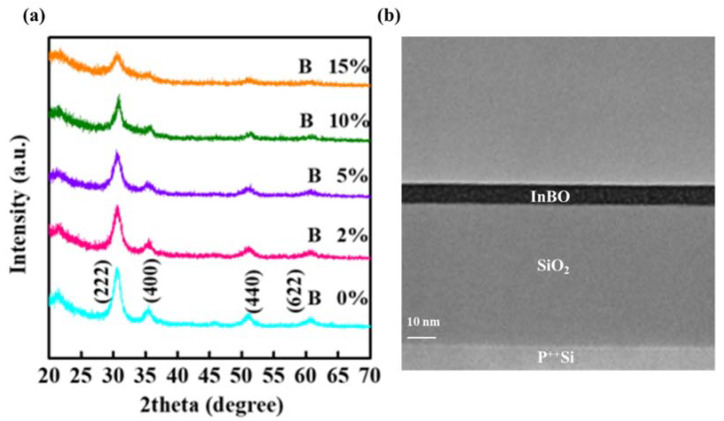
(**a**) GIXRD patterns obtained for In-B-O thin films with different B ratio; (**b**) the TEM cross-sectional view of In-B-O (10% B) layer.

As shown in Figure 1a, GIXRD is used to investigate the microstructure of In-B-O films with different B incorporations. It can be confirmed that In_2_O_3_ is a polycrystalline structure. The main orientations (222) of intrinsic In_2_O_3_ thin film appear at 2θ = 31.1°. The crystallite size of the In-B-O film is extracted from the following expression:

(1) $$D=0.94\;\frac{\lambda }{B\cos\left(\theta \right)}$$

where λ represents the wavelength of incident radiation, B denotes the diffraction peak at full width at half-maximum (FWHM) and θ is the Bragg angle corresponding to the selected XRD peak [11].

It can be calculated that the grain sizes of 0, 2, 5, 10 and 15% B-doped In_2_O_3_ are 9.903, 8.146, 6.802, 6.539 and 5.756 nm, respectively. Moreover, the reflection peaks of (222), (400), (440) and (622) are weakened with the increase in the B component, suggesting the reduction of crystallinity. However, the characteristic peaks correspond to the cubic In_2_O_3_ structure, and no other phases are found in the pattern, which means that the B doping does not destroy the In_2_O_3_ lattice properties, while the addition of B may be able to replace the In sites and maintain the cubic In_2_O_3_ structure [11,21]. To illustrate the microstructural characteristics of In-B-O, cross-sectional TEM were performed on the 10% B-doped In-B-O film based on the SiO_2_/Si substrate. As shown in Figure 1b, well-defined layers with uniform thicknesses (~10 nm) across the samples could be observed. The high-quality In-B-O/SiO_2_ interface could ensure high levels of electrical performance.

**Figure 2:**
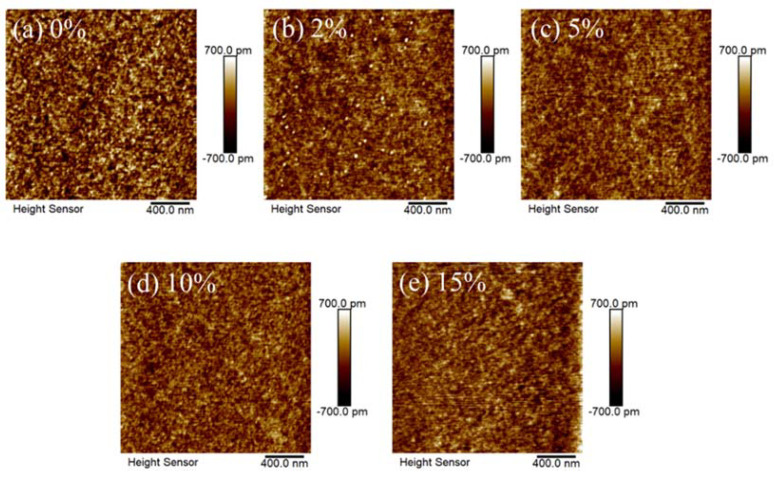
AFM images of In-B-O layers with specified B doping ratio of (**a**) 0%, (**b**) 2%, (**c**) 5%, (**d**) 10% and (**e**) 15%.

The AFM images of In-B-O layers with various B ratios are presented in Figure 2. The root-mean-square (RMS) roughness of In-B-O films with 0, 2, 5, 10 and 15% B are 0.219, 0.175, 0.162, 0.150 and 0.185 nm, respectively. Note that all the In-B-O films are excellently smooth, which is in good agreement with the cross-sectional TEM results shown in Figure 1b. In addition, the excellent RMS also suggests the absence of large grains or prominent grain boundaries for the In-B-O films, which is in line with the GIXRD results shown in Figure 1a. It is rather surprising that nanometer-thin crystalline ternary oxide with an atomically smooth nature could be realized using a simple one-step spin-coated approach. The growth mechanism of such high-quality thin films has been reported in previous work by Kelso et al., which is also used in chemical vapor deposition and liquid phase epitaxy [22]. We think the model developed by Kelso et al. could be used to explain the high-quality low-roughness surfaces obtained with our method. However, the details of the growth mechanism still needs further investigations.

**Figure 3:**
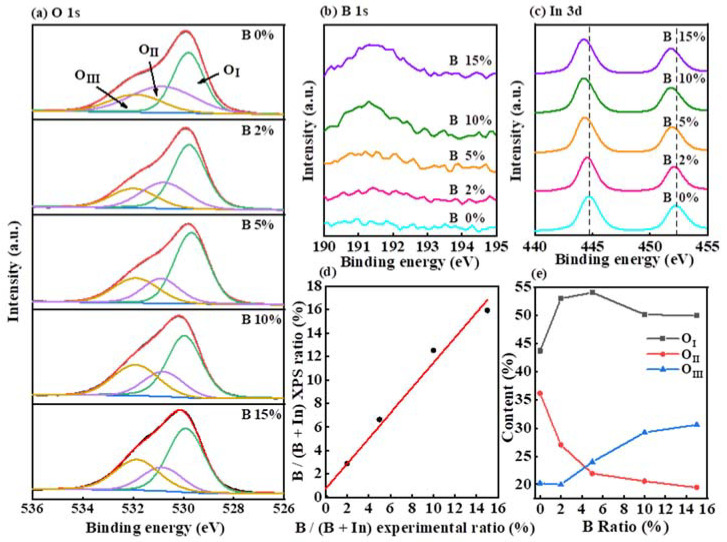
(**a**) The O 1s, (**b**) B 1s and (**c**) In 3d XPS spectra of In-B-O thin films with virous B ratios. (**d**) The atomic ratio diagram of B/(B+In) between precursor and film. (**e**) The relationship between the relative contents of O_I_, O_II_ and O_III_ with indicated B ratio.

The chemical states and compositions of In-B-O films were next analyzed via XPS. Figure 3a depicts the XPS spectra of the O 1s region of In-B-O films with various B contents. To discuss the changes in oxygen state, the XPS O 1s peak is fitted by three independent sub-peaks, representing the lower energy peak (O_I_) corresponds to lattice oxygen ions, which have neighboring In and B ions. The higher energy peak (O_II_) is related to oxygen from a hydroxide species present on the surface and in the films. The highest energy peak (O_III_) can be assigned to oxygen from water and carbonate present on the surface and in the film [23,24]. Via calculation, the O_II_/(O_I_+O_II_+O_III_) ratio with 0, 2, 5, 10 and 15% B are 36.13%, 27.01%, 21.93%, 20.58% and 19.42%, respectively (Figure 3e). It turns out that B plays an essential role in suppressing the related defects, such as the presence of hydroxide species. To further probe the effect of B content in In_2_O_3_ film, the corresponding XPS spectra of the In 3d with various B doping contents are shown in Figure 3b. It can be observed that the local environment remains almost unchanged with increasing B doping concentrations. However, the In peak gradually shifts toward low binding energy, which is, on the one hand, due to due to the bonding strength of B-O (808 kJ/mol) being stronger than that of In-O (346 kJ/mol), and on the other hand because B doping can reduce oxygen vacancy-related defects, resulting in a denser atomic stacking and enhanced electron shielding between adjacent atoms [25,26,27]. Figure 3c presents the XPS spectra of B 1s with different B contents. The apparent peak centered at 191.1 eV is related to the bonding of B_2_O_3_, revealing the formation of B-O bonds with increasing B content. Moreover, as shown in Figure 3d, it can be observed that the B ratio in the thin film is very close to the nominal synthetic concentration, proving that B has been incorporated into the In_2_O_3_ film.

**Figure 4:**
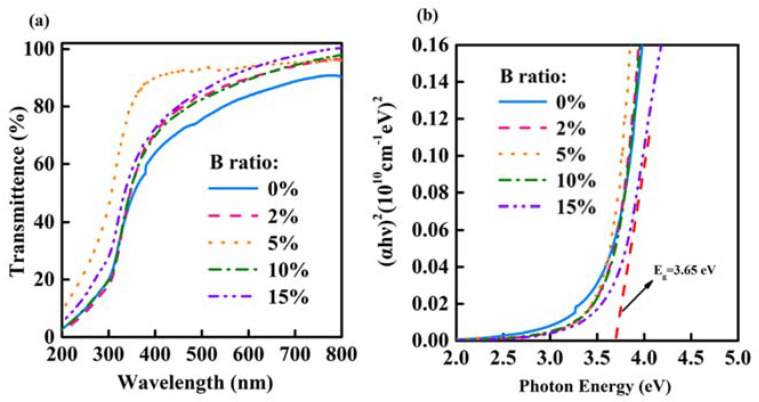
(**a**) The transmittance spectra and (**b**) Tauc plots of In-B-O films with indicated B ratio.

The effect of B doping on optical properties is illustrated in Figure 4a. All films show good transmission between 400 nm and 800 nm, illustrating that In-B-O has the potential to be used for transparent electronics. Figure 4b depicts the optical band gap (E_g_) of the In-B-O films. The value of E_g_ is extrapolated to the x-axis intercept by the slope of the curve, obtained using Tauc’s formula: (αℎν)^2^ = *C*(ℎν − *E*_*g*_). The E_g_ of In-B-O film with 0, 2, 5, 10 and 15% B is 3.42, 3.55, 3.56, 3.58 and 3.65 eV, respectively. The enhancement of E_g_ is attributed to bandgap of B_2_O_3_ (8.0 eV) being larger than that of In_2_O_3_ (~3.61 eV) [14]. In addition, the oxygen vacancy defects in the oxide thin film can generate localized states in the bandgap, resulting in a decrease in the bandgap [28,29]. B doping increases the bandgap energy, proving that B can suppress the formation of oxygen vacancy defects in In_2_O_3_.

**Figure 5:**
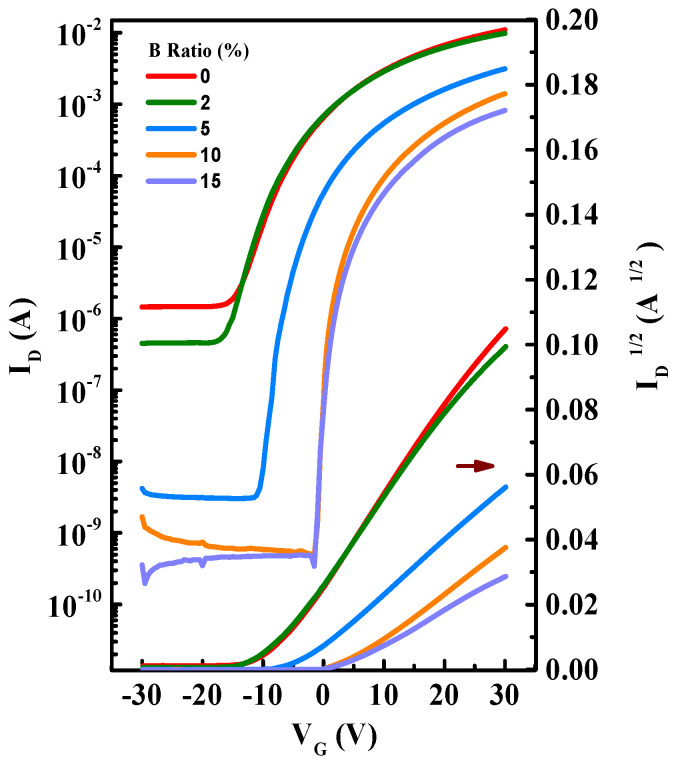
The transfer characteristics of In-B-O TFTs with various B contents.

**Figure 6:**
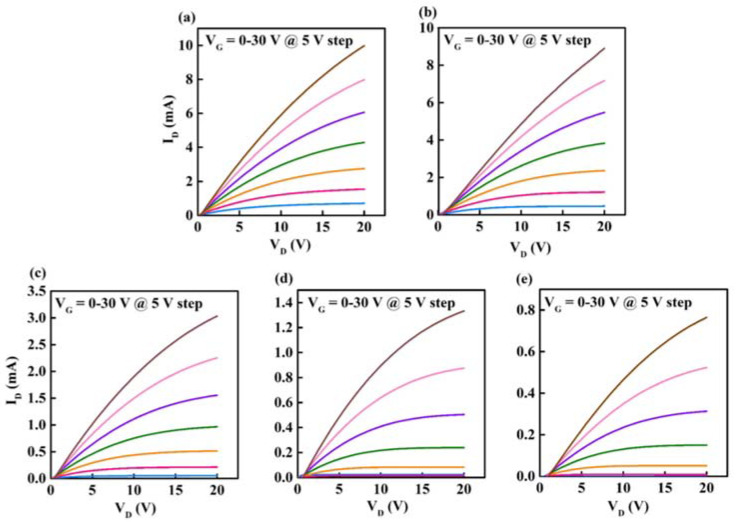
The output curves of In-B-O TFTs with indicated B ratios of (**a**) 0%, (**b**) 2%, (**c**) 5%, (**d**) 10% and (**e**) 15%.

**Figure 7:**
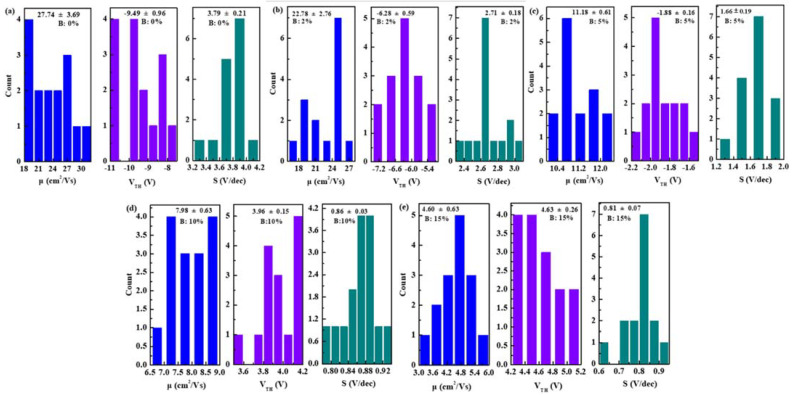
Electrical parameters histograms of μ, V_TH_ and S for In-B-O TFTs with B ratios of (**a**) 0%, (**b**) 2%, (**c**) 5%, (**d**) 10% and (**e**) 15%.

To validate the electrical properties of devices, the transfer and output characteristics of In-B-O TFTs are illustrated in Figure 5 and Figure 6, with detailed operating parameters exhibited in Table 2. The average values of mobility (μ), threshold voltage (V_TH_) and subthreshold swing (S) are gathered from 15 devices and shown in Figure 7, suggesting the good repeatability of the process. Without B doping, the oxide TFTs present high off-state current and negative V_TH_ values due to excess carrier concentrations, resulting in huge power consumption. As the B atom% changes from 0% to 10%, the off-state current drops significantly from 10^−6^ A to 10^−10^ A, resulting in on/off current ratio (I_on_/I_off_) increases from 7.57 × 10^3^ to 2.84 × 10^6^. Moreover, the V_TH_ shifts positively from −9.49 ± 0.96 V to 3.96 ± 0.15 V and S decreases from 3.79 ± 0.21 V/dec to 0.86 ± 0.03 V/dec. The improvement is due to the inhibition of the oxygen vacancy-related defects after B doping. However, B incorporation would decrease the In 5s orbital interaction near the conduction band edge, and hence reduce the mobility [16]. Therefore, the μ decreases from 27.74 ± 3.69 cm^2^/(V s) to 7.98 ± 0.63 cm^2^/(V s) as the B ratio increases from 0% to 10%, which is still sufficient for AMLCD applications. The high Lewis acid strength of B could alleviate dopant-induced carrier scattering and maintain the In_2_O_3_-based material mobility at a relatively high level [18]. Furthermore, the μ further reduces to 4.60 ± 0.63 cm^2^/(V s) for 15% B, owing to further decrease in the In 5s orbital interaction and/or the impurity (B(OH)_3_) scattering. We find that the 10% B ratio is the optimal composition for an In-B-O device, which shows a μ of 7.98 ± 0.63 cm^2^/(V s), an I_on_/I_off_ of 2.84 × 10^6^, a V_TH_ of 3.96 ± 0.15 V and an S of 0.86 ± 0.03 V/dec. Figure 6 presents the output characteristic of In-B-O TFTs, and all the devices exhibit the linear increase at small V_DS_, indicating the good interface contacts between the Al electrodes and the In-B-O active materials.

**Figure 8:**
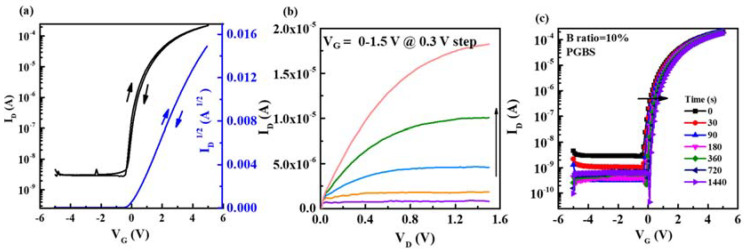
(**a**) The typical transfer and (**b**) output curves of solution-processed In-B-O /ZrO_2_ TFTs; (**c**) transfer characteristics evolution of In-B-O/ZrO_2_ TFTs under PGBS (4 V) for a duration of 1440 s.

The optimized In-B-O was further integrated with an aqueous solution-processed high-K ZrO_2_ dielectric to enhance the device performance and demonstrate the advantage of all aqueous solution processing. Water has been shown to be a superb solvent, since it does not contain organic residues that would need to be removed. Furthermore, nitrated salts in water usually form hexaaqua structures (M(H_2_O)_6_, where M denotes In, B or Zr) that could be easily broken with a low energy supply and yield dense and smooth oxide thin films. Figure 8a,b show the transfer and output curves of the fully water-processed In-B-O (10% B)/ZrO_2_ TFTs. Here, we extracted the electrical performance of devices, affording a μ of 11.0 cm^2^/(V s), an I_on_/I_off_ of ~10^5^, a V_TH_ of 0.09 V and an S of 0.19 V/dec. Furthermore, the device could be operated at a low operating voltage of 3 V, making it suitable for low-power applications. Moreover, to estimate the electrical stability of the In-B-O/ZrO_2_ TFTs, positive gate bias stress (PGBS) is performed with a V_G_ of 4 V for 1400 s. A small positive V_TH_ shift could be observed (due to electrons at the channel/dielectric interface), suggesting the superior stability of In-B-O/ZrO_2_ TFTs. The high performance of the In-B-O/ZrO_2_ TFTs could be ascribed to the high capacitance of the ZrO_2_ dielectric (~150 nF/cm^2^), the high quality of the In-B-O channel and excellent channel/dielectric interface. It is worth nothing that the high-performance In-B-O/ZrO_2_ device could be made using a one-pot aqueous solution at a moderate processing temperature, giving it great potential for use in cost-effective and large-scale green electronics.

Through the above characterization, we found that B incorporation could suppress oxygen vacancy-/hydroxyl-related defects due to the high B-O bonding dissociation energy, and hence improve the device performance. It is revealed that the simple water process could achieve an In-B-O with an ultrasmooth surface and a high-quality channel/insulator interface, which contributes to excellent device performance. In addition, the ultrathin crystalline nature of In-B-O could reduce the number of total defect states in the channel. Note that Young’s modulus is inversely proportional to thin film thickness, and the ultrathin In-B-O could also withstand high mechanical strain for flexible device [11].

## 4. Conclusions

In summary, we have demonstrated the creation of an ultrathin In-B-O TFTs using a facile, eco-friendly solution process. This approach enables the fabrication of high-quality crystalline In-B-O with an atomically smooth surface. Moreover, the B doping ratio could be tuned easily by the aqueous precursor solution. The role of B doping on the physical, chemical and electrical properties of the In_2_O_3_ is intensively investigated. The results show that B doping can suppress oxygen vacancy-/hydroxyl-related defects and alleviate dopant-induced carrier scattering, which is ascribed to the large metal–oxide bonding dissociation and high Lewis acid strength of B. Electrical measurements show that the incorporation of B can effectively improve the I_on_/I_off_, V_TH_ and S values of pristine In_2_O_3_ devices. The optimized In-B-O device on Si/SiO_2_ shows a μ of 7.98 ± 0.63 cm^2^/(V s), an I_on_/I_off_ of 2.84 × 10^6^, a V_TH_ of 3.96 ± 0.15 V and an S of 0.86 ± 0.03 V/dec. Furthermore, fully water-derived In-B-O/ZrO_2_ TFTs exhibit a μ of 11.0 cm^2^/(V s), a V_TH_ of 0.09 V, an I_on_/I_off_ of ~10^5^, an S of 0.19 V/dec, a low operating voltage of 3 V and superior stability. Therefore, an ultrathin crystalline In-B-O produced using a simple one-step spin-coating method and a water-processed method is considered to be a promising channel material for future low-cost and large-area advanced oxide electronics.

## Figures and Tables

**Table 1:** Electronegativity, metal–oxygen bonding strength and Lewis acid strength of elements.

Elements	Metal–Oxide Bonding Dissociation Energy (KJ/mol)	Lewis Acid Strength
In^3+^	320.1	1.026
Ga^3+^	353.5	1.167
Ba^2+^	502.9	1.163
Mg^2+^	363.2	1.402
Al^3+^	511.0	3.042
La^3+^	799.0	0.852
Sr^2+^	549.5	1.417
Y^3+^	719.6	1.465
Gd^3+^	719.0	0.788
Sc^3+^	681.6	1.697
Zr^4+^	776.1	2.043
Hf^4+^	801.7	1.462
Ti^4+^	672.4	3.064
Nb^5+^	771.8	2.581
Si^4+^	799.6	8.096
Ta^5+^	799.1	1.734
B^3+^	808.8	10.709

**Table 2:** Electrical properties of In-B-O TFTs with different B doping concentrations.

B Doping Ratios (%)	μ (cm^2^/Vs)	I_on_/I_off_	V_TH_ (V)	S (V/dec)
0	27.74 ± 3.69	7.57 × 10^3^	−9.49 ± 0.96	3.79 ± 0.21
2	22.78 ± 2.76	2.20 × 10^4^	−6.28 ± 0.59	2.71 ± 0.18
5	11.18 ± 0.61	1.04 × 10^6^	−1.88 ± 0.16	1.66 ± 0.19
10	7.98 ± 0.63	2.84 × 10^6^	3.96 ± 0.15	0.86 ± 0.03
15	4.60 ± 0.63	4.19 × 10^6^	4.63 ± 0.62	0.81 ± 0.07

## References

[1] Kamiya T., Hosono H. (2010). Material characteristics and applications of transparent amorphous oxide semiconductors. NPG Asia Mater..

[2] Fortunato E., Barquinha P., Martins R. (2012). Oxide semiconductor thin-film transistors: A review of recent advances. Adv. Mater..

[3] Hwan Hwang Y., Seo J.-S., Moon Yun J., Park H., Yang S., Ko Park S.-H., Bae B.-S. (2013). An ‘aqueous route’ for the fabrication of low-temperature-processable oxide flexible transparent thin-film transistors on plastic substrates. NPG Asia Mater..

[4] Jeong S., Moon J. (2012). Low-temperature, solution-processed metal oxide thin film transistors. J. Mater. Chem..

[5] Kim Y.H., Heo J.S., Kim T.H., Park S., Yoon M.H., Kim J., Oh M.S., Yi G.R., Noh Y.Y., Park S.K. (2012). Flexible metal-oxide devices made by room-temperature photochemical activation of sol-gel films. Nature.

[6] Xu W., Wang H., Xie F., Chen J., Cao H., Xu J.B. (2015). Facile and environmentally friendly solution-processed aluminum oxide dielectric for low-temperature, high-performance oxide thin-film transistors. ACS Appl. Mater. Interfaces.

[7] Li S., Tian M., Gao Q., Wang M., Li T., Hu Q., Li X., Wu Y. (2019). Nanometre-thin indium tin oxide for advanced high-performance electronics. Nat. Mater..

[8] Si M., Hu Y., Lin Z., Sun X., Charnas A., Zheng D., Lyu X., Wang H., Cho K., Ye P.D. (2021). Why In_2_O_3_ Can Make 0.7 nm Atomic Layer Thin Transistors. Nano Lett..

[9] Xu W., Li H., Xu J.B., Wang L. (2018). Recent Advances of Solution-Processed Metal Oxide Thin-Film Transistors. ACS Appl. Mater. Interfaces.

[10] Yu X., Marks T.J., Facchetti A. (2016). Metal oxides for optoelectronic applications. Nat. Mater..

[11] Li Y., Zhu D., Xu W., Han S., Fang M., Liu W., Cao P., Lu Y. (2020). High-mobility nanometer-thick crystalline In–Sm–O thin-film transistors via aqueous solution processing. J. Mater. Chem. C.

[12] Jaehnike F., Pham D.V., Bock C., Kunze U. (2019). Role of gallium and yttrium dopants on the stability and performance of solution processed indium oxide thin-film transistors. J. Mater. Chem. C.

[13] Zhang X., Wang B., Huang W., Chen Y., Wang G., Zeng L., Zhu W., Bedzyk M.J., Zhang W., Medvedeva J.E. (2018). Synergistic Boron Doping of Semiconductor and Dielectric Layers for High-Performance Metal Oxide Transistors: Interplay of Experiment and Theory. J. Am. Chem. Soc..

[14] Stewart K.A., Gouliouk V., Keszler D.A., Wager J.F. (2017). Sputtered boron indium oxide thin-film transistors. Solid-State Electron..

[15] Lin Z., Lan L., Sun S., Li Y., Song W., Gao P., Song E., Zhang P., Li M., Wang L. (2017). Solution-processed high-mobility neodymium-substituted indium oxide thin-film transistors formed by facile patterning based on aqueous precursors. Appl. Phys. Lett..

[16] Lee S.-H., Kim T., Lee J., Avis C., Jang J. (2017). Solution-processed gadolinium doped indium-oxide thin-film transistors with oxide passivation. Appl. Phys. Lett..

[17] Parthiban S., Kwon J.-Y. (2015). Amorphous boron–indium–zinc-oxide active channel layers for thin-film transistor fabrication. J. Mater. Chem. C.

[18] Parthiban S., Kwon J.-Y. (2014). Role of dopants as a carrier suppressor and strong oxygen binder in amorphous indium-oxide-based field effect transistor. J. Mater. Res..

[19] Park H., Nam Y., Jin J., Bae B.-S. (2014). Improvement of bias stability of oxyanion-incorporated aqueous sol–gel processed indium zinc oxide TFTs. J. Mater. Chem. C.

[20] Kim B.K., On N., Choi C.H., Kim M.J., Kang S., Lim J.H., Jeong J.K. (2021). Polycrystalline Indium Gallium Tin Oxide Thin-Film Transistors With High Mobility Exceeding 100 cm^2^/(V s). IEEE Electron Device Lett..

[21] Li Y., Xu W., Liu W., Han S., Cao P., Fang M., Zhu D., Lu Y. (2019). High-Performance Thin-Film Transistors with Aqueous Solution-Processed NiInO Channel Layer. ACS Appl. Electron. Mater..

[22] Kelso M.V., Mahenderkar N.K., Chen Q., Tubbesing J.Z., Switzer J.A. (2019). Spin coating epitaxial films. Science.

[23] Du X., Flynn B.T., Motley J.R., Stickle W.F., Bluhm H., Herman G.S. (2014). Role of Self-Assembled Monolayers on Improved Electrical Stability of Amorphous In-Ga-Zn-O Thin-Film Transistors. ECS J. Solid State Sci. Technol..

[24] Rajachidambaram J.S., Sanghavi S., Nachimuthu P., Shutthanandan V., Varga T., Flynn B., Thevuthasan S., Herman G.S. (2012). Characterization of amorphous zinc tin oxide semiconductors. J. Mater. Res..

[25] Hong L., Xu W., Liu W., Han S., Cao P., Fang M., Zhu D., Lu Y. (2020). High performance indium dysprosium oxide thin-film transistors grown from aqueous solution. Appl. Surf. Sci..

[26] Ting C.-C., Fan H.-Y., Tsai M.-K., Li W.-Y., Yong H.-E., Lin Y.-F. (2014). Improvement of electrical characteristics in the solution-processed nanocrystalline indium oxide thin-film transistors depending on yttrium doping concentration. Phys. Status Solidi A.

[27] Zhang Y., He G., Wang L., Wang W., Xu X., Liu W. (2022). Ultraviolet-Assisted Low-Thermal-Budget-Driven alpha-InGaZnO Thin Films for High-Performance Transistors and Logic Circuits. ACS Nano.

[28] Kumar S.S., Rubio E.J., Noor-A-Alam M., Martinez G., Manandhar S., Shutthanandan V., Thevuthasan S., Ramana C.V. (2013). Structure, Morphology, and Optical Properties of Amorphous and Nanocrystalline Gallium Oxide Thin Films. J. Phys. Chem. C.

[29] Anderson O.L., Schreiber E. (1965). The relation between refractive index and density of minerals related to the Earth’s mantle. J. Geophys. Res..

